# Genetic and environmental contributions to the link between synaesthesia and neurodevelopmental and psychiatric features: a twin study

**DOI:** 10.1038/s41398-025-03444-x

**Published:** 2025-07-12

**Authors:** Janina Neufeld, Tessa M. van Leeuwen, Ralf Kuja-Halkola, Sebastian Lundström, Henrik Larsson, Paul Lichtenstein, Sven Bölte, David Mataix-Cols, Mark J. Taylor

**Affiliations:** 1 Center of Neurodevelopmental Disorders at Karolinska Institutet (KIND), Department of Women’s and Children’s Health, Centre for Psychiatry Research, Karolinska Institutet and Stockholm Health Care Services, Region Stockholm, Stockholm, Sweden; 2 Swedish Collegium for Advanced Study, Uppsala, Sweden; 3 Tilburg School of Humanities and Digital Sciences, Department of Communication and Cognition, Tilburg University, Tilburg, the Netherlands; 4 Donders Institute for Brain, Cognition and Behaviour, Radboud University, Nijmegen, the Netherlands; 5 Department of Medical Epidemiology and Biostatistics, Karolinska Institutet, Stockholm, Sweden; 6 Gillberg Neuropsychiatry Centre, University of Gothenburg, Gothenburg, Sweden; 7 School of Medical Sciences, Örebro University, Örebro, Sweden; 8 Curtin Autism Research Group, Curtin School of Allied Health, Curtin University, Perth, WA Australia; 9 Child and Adolescent Psychiatry, Stockholm Health Care Services, Region Stockholm, Stockholm, Sweden; 10 Department of Clinical Neuroscience, Centre for Psychiatry Research, Karolinska Institutet and Stockholm Health Care Services, Region Stockholm, Stockholm, Sweden; 11 Department of Clinical Sciences, Lund University, Lund, Sweden

**Keywords:** Human behaviour, Psychiatric disorders

## Abstract

Synaesthesia is a sensory phenomenon where specific inputs such as written letters or tastes automatically trigger additional sensations (for instance colours). The phenomenon is more common in people on the autism spectrum compared to the general population and seems also to be associated with other neurodevelopmental and psychiatric conditions and features. We assessed the associations between self-reported synaesthesia and eight psychiatric / neurodevelopment features in 18-year-old twins and estimated the genetic and environmental contributions to these associations using classical twin modelling. All of the neurodevelopmental / psychiatric features (related to autism, ADHD, obsessive-compulsive disorder, anxiety, depression, psychotic-like experiences, eating disorders, and (hypo-)mania) correlated positively with self-reported synaesthesia. The strongest association was found with obsessive-compulsive features (r = 0.28). Genetic factors explained more than 50% of most these associations. Environmental factors that are not shared by twins (non-shared environment) influenced the associations to different degrees, while the influence of environmental factors that are shared by twins was estimated to be negligible. Rather than being specifically linked to autism, synaesthesia seems to be associated with a wider range of neurodevelopmental / psychiatric features, and especially obsessive-compulsive features. Genetic factors play a predominant role in most of these associations, suggesting that synaesthesia might share part of its genetic causes with several neurodevelopmental / psychiatric conditions.

## Introduction

Synaesthesia is a perceptual phenomenon where certain sensory inputs or concepts automatically trigger consistent, internally generated sensations. For instance, music or tastes can trigger the experience of dynamically evolving coloured shapes or each letter of the alphabet is experienced in its own specific colour. These sensory “concurrent” experiences can appear on an inner mental screen or projected into the outside world and are often vivid and individual for every synaesthete [1]. However, some trigger-concurrent combinations are more common than expected by chance (for instance, the English letter A tends to be more commonly red), suggesting that linguistic properties play a role in the manifestation of trigger-concurrent couplings [2]. In adults, the prevalence of synaesthesia has been estimated to about 4% [3], but since more synaesthesia types are continuously discovered, the true prevalence of the phenomenon is likely higher.

Synaesthesia aggregates in families [4–6] and monozygotic (MZ) twins appear to share the trait more commonly than dizygotic (DZ) twins [7], suggesting that synaesthesia is at least partly heritable. In line with this hypothesis, we recently found self-reported synaesthesia to be 41–51% heritable, using classical twin modelling [8]. In addition, environmental factors unique to each twin (non-shared environment) contributed at least as much as genetic factors [8]. The shared environment, referring to factors shared by twins living in the same home, played no role. Taken together, the manifestation of synaesthesia appears to be both heritable and shaped by certain aspects of the environment.

Synaesthesia is more common in autistic compared to non-autistic individuals [9–11]. Further, the number of different synaesthesia types a synaesthete has and the degree to which people consistently choose similar colours for individual numbers and letters in a synaesthesia test correlate positively with autistic traits [12–15]. For several of these studies, these associations were restricted to, or more pronounced for, the autistic trait domain of attention to detail [12, 13, 15]. In line with these findings, self-reported synaesthesia correlated with self-reported autistic features in our recent twin study, and this correlation was stronger for non-social autistic features (a sub-set of features including attention to detail, repetitive behaviours and restricted interests) compared to social and communication autistic features [8]. Further, the association between self-reported synaesthesia and overall autistic features was predominantly (71%) attributed to genetic factors with the remaining covariance (29%) attributed to non-shared environmental factors [8]. That synaesthesia and autism spectrum conditions (henceforth referred to as “autism”), share some of their genetic makeup is consistent with a study where synaesthetes reported autism to be not only more common among themselves but also their first-degree relatives [16]. While the evidence for the link between synaesthesia and both autism diagnosis and autistic features is so far quite consistent, a study found no association between synaesthesia and autism polygenic scores [17].

Recent studies suggest that synaesthesia might co-occur with a wider range of neurodevelopmental and psychiatric conditions or features, although studies in this field are still sparse (see Table 1 for an overview). In a relatively recent large population-based study (*N* = 3742), people with objectively tested grapheme-colour synaesthesia (*n* = 95), where letters or numbers trigger specific colour sensations, more commonly self-reported to be diagnosed with not only autism, but also obsessive-compulsive disorder (OCD) and anxiety disorders [18]. However, only the association with anxiety disorders was replicated in a second experiment in a smaller sample, raising the question whether the association with OCD could have been introduced by a methodological bias [18] (please see the discussion for further details). In an independent study, however, self-reported obsessive-compulsive (OC) features have also been found to correlate with self-reported synaesthesia [19]. Further, children with grapheme-colour synaesthesia have been reported to have higher rates of anxiety disorders and to express both fewer positive and negative emotions than non-synaesthetic children [20].

**Table 1:** Literature-based evidence for associations between synaesthesia and DSM-5 diagnostic categories or related features.

Condition	Studies (first author and date)	Significant positive association	No statistically significant association
Autism Spectrum	Baron-Cohen et al. [11], Neufeld et al. [10], Hughes et al. [9], Carmichael et al. [18], Ward et al. [12, 13], Tilot et al. [17], Van Leeuwen et al. [60] & [15], Burghoorn et al. [14], Nugent et al. [16]	**11** (5 diagnosis, 6 features)	**2** (1 polygenic score, 1 self-rep. diagnosis)
OCD	Carmichael et al. [18], Wendler E [19]	**2** (1 self-rep. diagnosis, 1 features)	**1** (self-rep. diagnosis)
Anxiety	Carmichael et al. [18], Simner et al. [20]	**2** (1 self-rep. diagnosis, 1 features)	–
Depression	Carmichael et al. [18]		**1** (self-rep. diagnosis)
Schizophrenia / Schizotypy / Psychosis	Banissy et al. [21], Janik McErlean et al. [22], Simmonds-Moore et al. [23], Tilot et al. [17], Nugent et al. [16]	**4** (3 features, 1 polygenic score)	**1** (diagnosis)
PTSD	Hoffman et al. [61] & [24]	**2** (self-rep. diagnostic screening)	–
ADHD	Carmichael et al. [18]		**1** (self-rep. diagnosis)
Dyslexia	Carmichael et al. [18]		**1** (self-rep. diagnosis)
Insomnia	Carmichael et al. [18]		**1** (self-rep. diagnosis)

A more detailed version of this table can be found in the supplement (Supplementary Table 1). The bold values refer to the number of published scientific articles that have investigated the association between synaesthesia and the condition in question and found evidence for an association (second right most column) or did not find an statistically significant association (right most column).

Individuals with grapheme-colour synaesthesia were also found, at the group level, to have a slightly increased polygenic risk for schizophrenia [17]. Moreover, self-reported schizotypy features have been found to be associated with both self-reported and objectively tested synaesthesia [21–23]. Finally, veterans with objectively tested grapheme-colour synaesthesia fulfilled criteria for posttraumatic stress more often than veterans without grapheme-colour synaesthesia [24].

Taken together, there is still limited but growing evidence for synaesthesia to be associated with a wider range of both clinical neurodevelopmental and psychiatric diagnoses and sub-clinical neurodevelopmental and psychiatric features. These associations appear to depend on statistical power and study design and some conditions have rarely or never been assessed in association with synaesthesia.

For instance, self-reported diagnoses of ADHD and depression were only assessed for an association with synaesthesia in one previous study, where these associations were not significant [18]. However, ADHD frequently co-occurs with autism [25], and depression co-occurs with both, anxiety and autism [25], and hence, an association of these conditions with synaesthesia could be expected. Eating disorders and bipolar disorder have, to the best of our knowledge, never been tested for an association with synaesthesia, but also co-occur with autism [25]. Further, bipolar disorder is associated with psychotic symptoms during manic episodes [26] and is genetically correlated with schizophrenia [27].

In this study, we investigated the relationships between self-reported synaesthesia, i.e. a measure reflecting the amount of experienced synaesthesia types and how certain people are to experience a type of synaesthesia when it is described to them, and selected neurodevelopmental and psychiatric features. We chose those features related to conditions that either have been implicated to be associated with synaesthesia previously, or that are strongly linked to other conditions showing an association with synaesthesia. More specifically, we included features related to autism, OCD, anxiety, depression, schizophrenia, ADHD, eating disorders and bipolar disorder.

First, we assessed the relationships between self-reported synaesthesia and features related to neurodevelopmental and psychiatric conditions. Second, we estimated the genetic and environmental contributions to these associations using classical twin modelling.

The previously found co-occurrence and potentially shared aetiology of synaesthesia with neurodevelopmental and psychiatric conditions indicates that synaesthetes might have a to some degree increased likelihood / vulnerability to develop these conditions [28]. On the other hand, it needs to be pointed out that synaesthesia per se is not a disorder, and instead linked to advantages such as enhanced memory [29] and superiority in learning savant-like calendar calculations (earlier linked to autism) [30]. This study seeks to enhance our understanding of the potentially partially shared aetiology of synaesthesia as a non-pathological phenomenon with a range of conditions and features that are associated with difficulties in managing daily life. Ultimately, this might contribute to a better understanding of how burdening and beneficial non-normative variations in perception and cognition overlap.

## Materials and methods

### Participants

All participants were twins participating in the longitudinal Child and Adolescent Twin Study in Sweden (CATSS) [31]. At the age of 18 years, these twins completed a comprehensive online survey after giving their informed consent digitally, and for those born 1999 or later, the survey included a screening for different types of synaesthesia. Zygosity was assessed through a panel of 48 single-nucleotide polymorphisms where genetic data was available (48% of included twin pairs), via a 5-item parent-report questionnaire assessing twin similarity (45%) or concluded from opposite biological sex (7%). For same-sex twin pairs without genetically determined zygosity information where the questionnaire was inconclusive (i.e. there was less than 95% chance of correct classification) zygosity was deemed undetermined.

Out of *N* = 7610 twins who completed the CATSS18 survey including the synaesthesia screening, 864 individuals were excluded because more than one item of the synaesthesia screening was missing or answered with “I don’t know / don’t want to answer”. Further, 1588 individuals were excluded because their co-twin did not participate or was excluded due to missing data. In addition, 28 individuals were excluded due to undetermined zygosity. In total, 5144 individuals (2572 complete twin pairs) were included in the study. These twin pairs comprised 814 MZ pairs (513 female, 301 male), 923 same-sex DZ pairs (582 female, 341 male), and 835 opposite-sex DZ pairs. This sample was largely overlapping with the sample of our previous twin study on the association between synaesthesia and autistic features alone [7], except that in the current study sample was larger (882 additional participants) since an additional year of data collection had been completed. The study was performed in accordance with the Declaration of Helsinki and was approved by the Regional Ethical Review Authority in Stockholm (Dnrs: 2010/1410-31-1, 2018/141-32).

### Measures

#### Synaesthesia screening

After a brief introduction of synaesthesia, participants completed eight questions, inquiring whether, and at which level of confidence, they experienced different types of synaesthesia, including letter-colour, digit-colour, week-day-colour, month-colour, sound-colour, sequence-space, people-colour, and number-personality synaesthesia. Response possibilities were “yes” (scored as 1.0), “Yes, to some extent” (scored as 0.5), “No” (scored as 0) and “I don’t know / don’t want to answer” (coded as missing). For additional details, please see Taylor et al. [8].

#### Neurodevelopmental / psychiatric features

Continuous neurodevelopmental / psychiatric features were assessed via self-report within the same survey that included the synaesthesia screening. These comprised features related to autism, ADHD and obsessive-compulsive disorder, anxiety, depression, schizophrenia, eating disorders and bipolar disorder.

Like in our previous study [8], ***autistic features*** were assessed with the 12-item self-report version of the autism module of the Autism-Tics, AD/HD, and other Comorbidities inventory (A-TAC) [32, 33]. ***Obsessive-compulsive (OC) features*** were assessed using the Brief Obsessive-Compulsive Scale (BOCS), including 12 items [34]. ***Anxiety features*** were assessed with the 38-item Screen for Child Anxiety Related Emotional Disorders (SCARED) [35]. ***Depression-related features*** were assessed using the 11-item Center for Epidemiologic Studies Depression Scale (CES-D) [36]. ***Psychotic-like Experiences (PLE)***, as features linked to schizophrenia, were assessed using six out of the seven items of the Psychotic-like Experiences Scale [37]. One item on visual hallucinations (“Have you ever seen things that other people could not see?”) was excluded because it inquiries about experiences that could be confused with synaesthetic sensations. ***ADHD features*** were assessed using the 18-item Adult ADHD Self-Report Scale (ASRS) [38]. ***(Hypo-) mania-related features*** (features linked to bipolar disorder) were assessed with the 13-item Mood Disorder Questionnaire (MDQ) [39]. Finally, ***eating disorder-related features*** were assessed with the 22-item Eating Disorder Inventory - 2 (EDI-2) [40]. More details about the included measures can be found in the Supplementary Information, Section 1.

### Data analyses

All analyses were performed using R version 4.4.0, including the OpenMx package (version: 2.21.11; https://openmx.ssri.psu.edu/) for classical twin modelling.

#### Descriptive analyses and data preparation

Participants answering less than 80% of a scale (i.e. >20% missing values or responding “I don’t know / I don’t want to answer”) did not contribute to analyses involving the scale in question. This differed slightly between the variables (see Table 2), with 94% of the sample contributing to the analysis involving autistic features, almost 100% of the sample contributing to the analyses involving anxiety features and the percentages for the remaining measures lying in between these values. Variables were explored in terms of skewness, mean values and standard deviations. Internal consistency of each variable was explored by calculating Cronbach’s alpha. Internal consistency varied between acceptable and good (0.72–0.93) for all measures, except PLE where internal consistency was below 0.7, likely due to the small number of items.

**Table 2:** Descriptive statistics of included variables.

	nr. of items	n data (%)	skew (skew^a^)	α (95% CI)	mean (all / MZ / DZ)	sd (all / MZ / DZ)
Synaesthesia	8	5144 (100)	2.38 (1.14)	0.80 (0.80–0.81)	0.80 / 0.82 / 0.79	1.30 / 1.32 / 1.28
OC	12	5076 (98.7)	1.23 (0.16)	0.77 (0.77–0.78)	2.29 / 2.30 / 2.28	2.51 / 2.54 / 2.49
Anxiety	38	5124 (99.6)	0.96	0.93 (0.93–0.94)	18.33 / 18.81 / 18.10	12.56 / 12.76 / 12.46
Depression	11	5116 (99.5)	0.85	0.86 (0.85–0.87)	19.66 / 19.32 / 19.81	6.07 / 5.88 / 6.15
PLE	6	5131 (99.7)	2.70 (0.95)	0.65 (0.63–0.66)	0.98 / 1.04 / 0.95	1.57 / 1.66 / 1.53
ADHD	18	5140 (99.9)	0.66	0.92 (0.91–0.92)	24.89 / 24.30 / 25.16	12.92 / 13.13 / 12.81
Autism	12	4815 (93.6)	1.34 (0.02)	0.72 (0.71–0.73)	1.92 / 1.98 / 1.88	1.64 / 1.70 / 1.62
Eating	23	5121 (99.6)	0.87	0.93 (0.92–0.93)	32.11 / 31.68 / 32.30	20.38 / 20.06 / 20.53
(Hypo-)mania	13	4975 (96.7)	0.25	0.84 (0.84–85)	4.80 / 4.62 / 4.89	3.52 / 3.45 / 3.54

*n data* number of included participants with sufficient data per scale (% = percentage of total included sample), *sd* standard deviation, *α* Cronbach’s α, *95% CI* 95% confidence interval. Only variables with a skewness > 1 were log-transformed to reduce the skewness. *Autism* autistic features, *OC* obsessive-compulsive features, *Anxiety* anxiety features, *Depression* depression-related features, *PLE* psychotic-like experiences, *ADHD* ADHD features, *Eating* eating disorder-related features, *(Hypo-)mania* (Hypo-)mania-like symptoms.

^a^After log-transform.

Variables were log-transformed (*log(1* + *x)*) to reduce positive skew, where this was found to be greater than 1 and otherwise left untransformed. The synaesthesia screening score, autistic features, OC features and PLE were heavily skewed and hence log-transformed before further analyses (see Table 2). After that, each variable was regressed on birth year and sex in a separate model and the residuals were standardized and used for twin modelling analysis.

#### Main analyses

##### Correlation analyses

The associations between self-reported synaesthesia and the eight selected neurodevelopmental / psychiatric features were explored using correlation analyses (Pearson). Since the data from twins are not independent from each other, we randomly selected only one twin per pair for this correlation analyses. In order to identify the strongest of these correlations, we compared them using Pearson and Filon’s z for pair-wise comparisons of Pearson correlations (performed using the R-package cocor).

##### Bivariate quantitative genetic analyses

Before fitting the bivariate models, we tested the model assumptions that means and variances do not differ across twin order or zygosity. This was done for each of the included variables individually, fitting saturated and step-wise constrained saturated models (collapsing means and variances across twin order and zygosity groups). Subsequently, bivariate models, first fully saturated and then constrained by equal means and variances across twin order and zygosity groups were fitted.

From the constrained models, twin correlations for each phenotype and across the phenotypes (cross-twin cross-trait = CTCT) and their 95% confidence intervals were extracted.

We then used bivariate Cholesky decompositions, implemented as the mathematically equivalent correlated factors solutions, and ACE models were fitted in order to determine the bivariate heritability between differences in self-reported synaesthesia and each of the eight neurodevelopmental / psychiatric features. These models estimate the degree to which each of the modelled phenotypes can be attributed to additive genetic factors (A), environmental factors shared by twins (C) and environmental factors not shared by twins (E) and estimates the genetic and environmental correlations between the two modelled phenotypes. Subsequently, this information is used to estimate the relative contribution of each component (A, C, and E) to the phenotypic correlation between the two phenotypes. For each bivariate ACE model, the model fit was compared to the according fully saturated bivariate model.

Nested models of the ACE models where one or more variance components were excluded (AE, CE, E) were fitted to the data, and model fits of the nested models were compared to the model fit of the ACE model. This was done in order to determine whether any of the three variance components could be regarded as negligible in an association. If model fit of a nested model was not statistically significantly different from the ACE model, it was selected as the more parsimonious solution.

## Results

### Correlations between self-reported synaesthesia and neurodevelopmental / psychiatric features

**Fig. 1:**
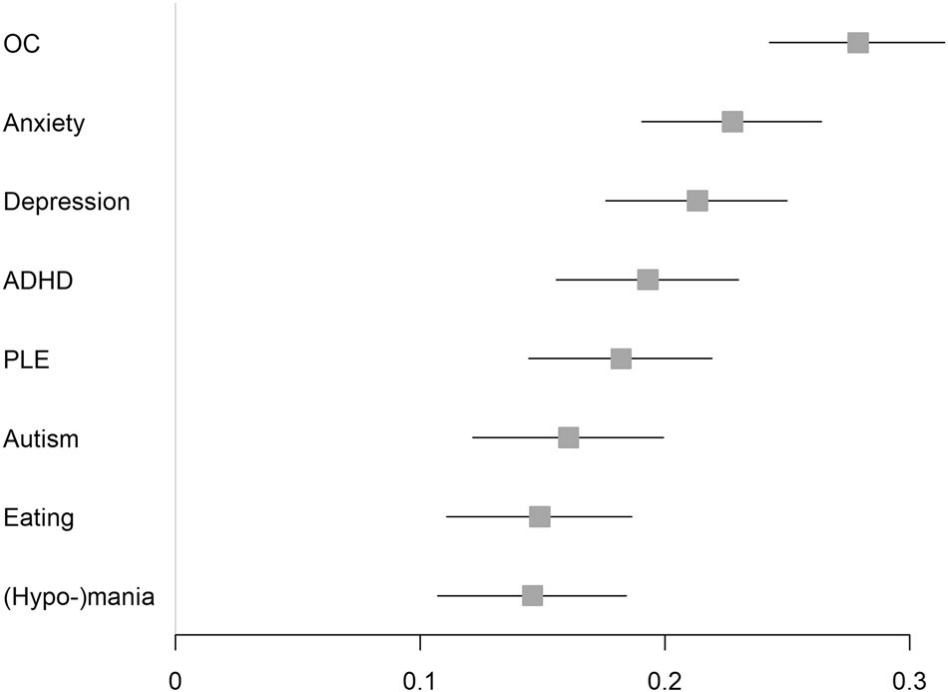
Forest plot. Pearson correlations between synaesthesia screening score and the selected neurodevelopmental / psychiatric trait measures in half of the sample (one randomly selected twin per pair). Boxes represent r-values and lines their 95% confidence intervals, calculated using the R-package cor.test (function = conf.int; method = “pearson”). Autism autistic features, OC obsessive-compulsive features, Anxiety anxiety features, Depression Depression-related features, PLE psychotic-like experiences, ADHD ADHD features, Eating Eating disorder-related features, (Hypo-)mania (Hypo-)mania-related features.

All of the neurodevelopmental / psychiatric features correlated positively (all *p*-values < 0.0001) with self-reported synaesthesia (see Fig. 1 and Supplementary Tables 2 & 3). These correlations were small, with the correlation with OC features being largest (r = 0.28). Note that all these correlations would survive a Bonferroni-correction for multiple comparisons, as all *p*-values were very small. The synaesthesia screening score correlated more strongly with OC features compared all of the other features (see Supplementary Table 4).

### Twin modelling results

The univariate model assumptions were met for all of the included variables except depression-related features (see Supplementary Table 5).

All of the CTCT correlations were larger in MZ twins than DZ twins (see Table 3). For OC features, the contrast between MZ and DZ CTCT was most pronounced (with r = 0.16 versus 0.05 in MZ and DZ, respectively).

**Table 3:** Phenotypic and CTCT correlations between synaesthesia screening score and neurodevelopmental / psychiatric features.

	rPh (95% CI)	rA (95% CI)	rE (95% CI)	CTCT MZ	CTCT DZ
OC	0.25 (0.22–0.27)	0.33 (0.25–0.41)	0.18 (0.13–0.24)	0.16 (0.12–0.20)	0.05 (0.02–0.08)
Anxiety	0.19 (0.16–0.21)	0.29 (0.21–0.37)	0.09 (0.03–0.15)	0.14 (0.10–0.18)	0.06 (0.02–0.09)
Depression	0.18 (0.15–0.21)	0.27 (0.19–0.35)	0.10 (0.05–0.16)	0.13 (0.09–0.17)	0.05 (0.02–0.08)
PLE	0.16 (0.14–0.19)	0.26 (0.17–0.34)	0.10 (0.04–0.15)	0.11 (0.07–0.15)	0.05 (0.01–0.08)
ADHD	0.16 (0.13–0.19)	0.25 (0.16–0.32)	0.07 (0.01–0.13)	0.12 (0.08–0.16)	0.07 (0.04–0.10)
Autism	0.18 (0.15–0.20)	0.27 (0.19–0.35)	0.10 (0.04–0.16)	0.12 (0.08–0.16)	0.07 (0.03–0.10)
Eating	0.09 (0.06–0.12)	0.07 (−0.01–0.15)	0.11 (0.05–0.17)	0.04 (0.00–0.08)	0.00 (−0.03–0.03)
(Hypo-)mania	0.16 (0.13–0.19)	0.21 (0.12–0.30)	0.12 (0.07–0.18)	0.10 (0.06–0.14)	0.03 (0.00–0.06)

Phenotypic (rPh), additive genetic (rA) and non-shared environmental correlations (rE) derived from bivariate AE models and phenotypic and cross-twin-cross trait (CTCT) correlations in sub-samples of monozygotic (MZ) and dizygotic (DZ) twins, extracted from the constrained, saturated bivariate models.

*CI* confidence interval, *Autism* autistic features, *OC* obsessive-compulsive features, *Anxiety* anxiety features, *Depression* depression-related features, *PLE* psychotic-like experiences, *ADHD* ADHD features, *Eating* eating disorder-related features, *(Hypo-)mania* (Hypo-)mania-like symptoms.

For all variables, the bivariate ACE-models had an acceptable fit to the data (see Supplementary Table 6). For the bivariate model assessing the association of self-reported synaesthesia and eating disorder-related symptoms, but not any of the other models, the model fit was worse for the ACE model compared to the saturated model. For completeness, the univariate twin correlations from bivariate models can be found in Supplementary Table 7 and univariate model results can be found in Supplementary Table 8.

In all bivariate ACE models, the shared environmental component C was estimated to be negligible, and hence all following results were extracted from AE models without the C component. The genetic correlation (rA) between synaesthesia and OC features was largest and can be considered as moderate (0.33) while all other genetic correlations were small (ranging between 0.07 and 0.27). All genetic correlations were larger than the respective non-shared environmental correlations (which ranged between 0.07 and 0.18, see Table 3).

The balance between additive genetic and non-shared environmental contributions differed between the models. Additive genetics were estimated to explain 38–78% of the correlations between self-reported synaesthesia and neurodevelopmental / psychiatric features and non-shared environment was estimated to explain 26–62%, see Table 4. More specifically, genetic influences (A) were estimated to be larger than shared environmental contributions (E) for all associations between self-reported synaesthesia and neurodevelopmental / psychiatric features except eating disorder-related features, and 95% confidence intervals were non-overlapping between A and E for associations with OC features, anxiety features, PLE and ADHD features.

**Table 4:** Covariance components.

	A (95% CI)	E (95% CI)
OC	0.74 (0.57–0.90)	0.26 (0.10–0.43)
Anxiety	0.70 (0.53–0.87)	0.30 (0.13–0.47)
Depression	0.66 (0.46–0.85)	0.34 (0.15–0.54)
PLE	0.78 (0.59–0.96)	0.22 (0.04–0.41)
ADHD	0.70 (0.52–0.88)	0.30 (0.12–0.48)
Autism	0.59 (0.46–0.72)	0.41 (0.28–0.54)
Eating	0.38 (0.01–0.75)	0.62 (0.25–0.99)
(Hypo-)mania	0.56 (0.35–0.77)	0.44 (23–0.65)

Variance components derived from the bivariate AE models. A = proportion of the phenotypic correlation that was attributed to additive genetics; E = proportion of the phenotypic correlation that was attributed to non-shared environment.

*CI* confidence interval, *Autism* autistic features, *OC* obsessive-compulsive features, *Anxiety* anxiety features, *Depression* depression-related features, *PLE* psychotic-like experiences, *ADHD* ADHD features, *Eating* eating disorder-related features, *(Hypo-)mania* (Hypo-)mania-like symptoms.

Together, the results suggest that, for most neurodevelopmental / psychiatric features, genetic influence dominates the association with synaesthesia, while non-shared environment contributes significantly but to a lesser degree.

## Discussion

### Phenotypic associations between self-reported synaesthesia and neurodevelopmental / psychiatric features

We found self-reported synaesthesia to be correlated to all of the investigated neurodevelopmental and psychiatric features. We expected that the correlation with autistic features would have been one of the strongest of these correlations, since the association between synaesthesia and autism is relatively well established. However, the strongest phenotypic (and genetic) correlation observed in this study was between self-reported synaesthesia with OC features, followed by anxiety features. These findings are in line with the first experiment in the study by Carmichael et al. [18], where self-reported diagnoses of anxiety, OCD and autism were found to be associated with objectively tested synaesthesia, and studies finding an association between OC features and self-reported synaesthesia [19] and anxiety and objectively tested synaesthesia in children [20]. Our results are, however, in contrast to the second experiment by Carmichael et al. [18] where the association with OCD was lost while the association with anxiety remained. The authors suggested that the result of the first experiment might have been biased by people with OCD being better at completing the long repetitive synaesthesia test compared to people without the condition. In their first experiment, only individuals indicating having synaesthesia were tested. In the second experiment, everyone completed the test, regardless of the self-report. If participants in the first experiment who disliked repetitive tasks guessed that they would be tested for synaesthesia when indicating having it, they might have negated it in order to avoid having to complete the test. The second experiment, however, lacked the power to assess the association between synaesthesia and OCD [18] and introduced two other biases the first experiment did not have. More specifically, the study was advertised as a synaesthesia study and the sample was predominantly (>90%) female. The discrepancies between the results of the two experiments indicate that findings of associations between synaesthesia and OCD (and potentially also other psychiatric conditions) are sensitive to study design. While it cannot be excluded that the results from Carmichael et al.’s [18] first experiment were biased, our results can at least not be affected by the same bias, since no repetitive test was performed. Overall, we believe that our study together with the previous studies suggest that there is indeed an association between synaesthesia and OCD/OC features. However, additional sufficiently powered studies are needed to confirm this association, ideally also for objectively tested synaesthesia and confirmed clinical OCD diagnosis. Since most measures used in this study are relatively brief with few items per sub-scale (e.g. the OC sub-scales have only 2–4 items each) we decided against looking into sub-scale specific associations. In order to better understand the link between synaesthesia and OCD/OC features, it would, however, be interesting to know whether specific OC feature domains drive this association, similar as previously observed for the association between synaesthesia and autistic features [8, 12, 13, 15].

Our results also strengthen previous evidence for an association between synaesthesia and anxiety [18, 20] and alterations in mood, where one previous study suggested an association between synaesthesia and the expression of both fewer positive and negative emotions in children [20] and our study suggests an association with depression- and (hypo-)mania-related features in young adults. Moreover, our findings suggest that self-reported synaesthesia is associated with a broad range of neurodevelopmental and psychiatric features, rather than being specifically linked to autism.

The question arises, why synaesthesia should be linked to a broad range of conditions or features. Synaesthesia has been found to be associated with a sensory processing profile that is highly similar to what is typical for people on the autism spectrum, including both hyper- and hypo-sensitivity to sensory stimuli, enhanced attention to detail and less automatic integration of sensory features into a global whole [41]. This led to the hypothesis that synaesthesia and autism share the part of their aetiology that is linked to altered sensory processing [41]. Some of these shared sensory features, namely sensory hyper- and sometimes even hypo-sensitivity, are also common among people with OCD, anxiety, depression, and ADHD [42–44], suggesting that they might be transdiagnostic features [43], shared between a wider range of neurodevelopmental and psychiatric conditions and synaesthesia [8]. In autism, sensory features have been proposed to play an important role in its overall aetiology [45], and their link to other autistic features has been estimated to be predominantly genetic [46]. The role of sensory features in other conditions is less explored and discussed. Since altered sensory processing appears to be an (often neglected) feature implicated in most conditions that we found to correlate with synaesthesia, we speculate that the associations between synaesthesia and neurodevelopmental and psychiatric features might be driven by transdiagnostic sensory features rather than synaesthesia leading to the development of psychiatric and neurodevelopmental conditions or vice versa. Alternatively, synaesthesia might, independently from altered sensory processing, be linked to a general factor of psychopathology [47].

Studies assessing associations between synaesthesia and conditions and features outside the autism spectrum are scarce to date. To the best of our knowledge, only the study by Carmichael et al. [18] simultaneously assessed synaesthesia in association to a wider range of conditions previously [26], finding partially similar results, but for instance no evidence for an association with ADHD and depression. Our study was methodologically complementary to Carmichael et al.’s [18] study, which might explain the discrepancies. For instance, associations with binary diagnostic categories, like in Carmichael et al.’s [18] study, compared to dimensional features, like in our study, can differ, with the former being more important to assess clinical relevance and the latter capturing more variance including subtle, sub-clinical features, making it the more sensitive approach.

Neurodevelopmental conditions are today seen as the extreme end of continuously distributed features or symptoms [48]. Psychiatric disorders and the according subclinical features share at least part of their genetic risk factors, supporting the idea that even psychiatric disorders are more likely to be continuous phenotypes rather than the categorical entities [49]. Ideally, future studies should use both categorical clinical and dimensional definitions in combination in order to gain a more complete understanding of the relationship between synaesthesia and mental health.

### Genetic and environmental contributions to the associations between self-reported synaesthesia and neurodevelopmental / psychiatric features

Our bivariate classical twin model results suggest that synaesthesia shares a common genetic basis with all of the assessed neurodevelopmental / psychiatric features. These shared genetic factors might be related to the previously mentioned general factor of psychopathology [47] and / or more specifically to the previously discussed transdiagnostic sensory features, potentially via alterations in how sensory brain networks develop, their organization (e.g. connectivity) and functioning (e.g. excitation-inhibition balance) [50]. All of the observed phenotypic associations were, to different degrees, also partially attributed to non-shared environmental influences, while the influence of shared environment was estimated to be zero. Non-shared environmental factors comprise all factors that make twins different from each other and can include events before and during birth, but also later during development. Possible scenarios how non-shared environmental factors might influence both the likelihood of developing synaesthesia and the development of neurodevelopmental and psychiatric features might be related to early life events (e.g. infections, trauma etc.). The latter can, for instance, alter or delay development, or lead to an increase in neural plasticity and re-organization of neural circuits. In line with this view, the emergence of synaesthesia might be explained by reduced experience-dependent pruning during early postnatal development [51]. Findings of a reduced specialization to discriminate one’s own species’ faces and familiar speech sounds over other species’ faces and foreign speech sounds in synaesthetes (reduced perceptual narrowing) supports this idea [51]. Further, there is evidence for hyper-connectivity of multiple brain regions in (adult) synaesthetes [52]. Altered pruning or other processes affecting brain connectivity in synaesthetes might, however, be driven by genetic factors. Consistent with this view, a study identified the involvement of genes crucial for axon formation in synaesthesia [53]. Interestingly, altered pruning has also been pointed out as a shared mechanism of autism, ADHD, and schizophrenia [54], and might hence be a (likely genetically determined) shared mechanism between synaesthesia to these conditions.

Differences in prenatal environments might also play a role in synaesthesia, neurodevelopmental / psychiatric conditions and features, and their associations. For instance, autism, OCD, ADHD, and schizophrenia are associated with decreased birth weight, independent from preterm birth and familial confounding, suggesting that growth restrictions in utero might play a role in these conditions [55, 56]. These can differ even between twins, for instance nutrition supply in utero can be more advantageous for one twin compared to the other [57]. The potential role of growth restrictions or other prenatal environmental factors on the development of synaesthesia remains, however, to date unexplored.

Overall, in the light of previous studies and theories, our results suggest that synaesthesia might share several genetic and environmental contributing factors with neurodevelopmental / psychiatric conditions and features, that might additionally interact in the course of development.

### Limitations

Our correlational analyses do not allow any conclusions regarding the directions of these associations. The E component in ACE models also includes measurement error and can hence not be differentiated from genuine non-shared environmental influences. Measurement error in self-reported synaesthesia or other self-report measures could for instance arise when participants interpret the screening questions in different ways, meaning that some people might over-report and others under-report synaesthesia / other features. In some previous studies, the proportion of self-reported synaesthetes who also fulfilled the objective test criteria for synaesthesia was less than 50% [18, 58], indicating that over-reporting of synaesthesia is common (or that not all synaesthetes have consistent trigger-concurrent couplings). Finally, the current sample might have been underpowered for detecting small shared environmental components, which is a common problem in classical twin modelling [59]. However, the C component was estimated to be zero or very close to zero in all models (estimates below 0.02%), which suggests that it was indeed negligible rather than just not significant. Since CATSS is a population-based twin study, we assume that the prevalence of neurodevelopmental and psychiatric diagnoses in our sample reflects the prevalence in the general population. We did, however, not have access to recent diagnostic status information of this sample and were hence unable to confirm this assumption. For a comparison of levels of neurodevelopmental / psychiatric features in this and previous population-based studies, please see Supplementary Table 9.

## Conclusions

Our study was the first to estimate genetic and environmental contributions to associations between self-reported synaesthesia and a wide range of neurodevelopmental / psychiatric features and symptoms. In line with emerging previous evidence, our results indicate that synaesthesia is not specifically linked to autism, but a wider range of neurodevelopmental / psychiatric features and especially OC features. Genetic factors, possibly liked to transdiagnostic sensory features, and non-shared environmental factors, such as early life events that modulate brain organization, seem to drive these associations. Future studies should investigate potentially overlapping variations in genes that are crucial for sensory neural network development and explore the role of early life events such as trauma and infections.

## Supplementary information

Supplementary information for the article “Genetic and environmental contributions to the link between synaesthesia and neurodevelopmental and psychiatric features: A twin study.”

## Data Availability

The data are not publicly available but slightly modified versions of the variables (adding a small random noise) can be shared upon request. The modification of the data is necessary because according to the ethical and legal rules for data protection that apply for this study, only truly anonymized data can be shared publicly. Since the score combination of twin pairs could in principle be used to trace back individuals in our original data which the Swedish Twin Registry could link back to identifying information such as names or contact details, the data used in this study need to be regarded as pseudomized. Adding a small random noise variable to each interest variable separately does not change the results at large, although small differences in estimates or *p*-values several digits after the comma might occur. The code used for univariate and bivariate twin modelling is available under https://github.com/JaninaNeufeld/Synesthesia_Classical_Twin_Analysis.git.
