# Supplementary material for: Genetic and environmental contributions to the link between synaesthesia and neurodevelopmental and psychiatric features: a twin study
Source: Transl Psychiatry. 2025 Jul 12;15:240. doi: 10.1038/s41398-025-03444-x (PMC12254358; doi:10.1038/s41398-025-03444-x)
Supplement: Supplementary file 1 — Supplementary information for the article “Genetic and environmental contributions to the link between synaesthesia and neurodevelopmental and psychiatric features: A twin study.” [file 41398_2025_3444_MOESM1_ESM.pdf]

Supplementary information for the article

"Genetic and environmental contributions to the link between synaesthesia and neurodevelopmental and psychiatric features: A twin study."

Janina Neufeld, Tessa M. van Leeuwen, Ralf Kuja-Halkola, Sebastian Lundström, Henrik Larsson, Paul Lichtenstein, Sven Bölte, David Mataix-Cols, Mark J. Taylor

Content

1) ***Additional information about the selected neurodevelopmental / psychiatric feature variables***__________p. 2
2) ***Control variable Results***__________ p. 4
3) ***Supplementary Tables***
   Supplementary Table 1: More detailed version of Table 1 on previous findings regarding synaesthesia and neurodevelopmental and psychiatric conditions_____p. 5
   Supplementary Table 2: Pearson correlations between synaesthesia screening score and neurodevelopmental / psychiatric features__________p. 6
   Supplementary Table 3: Full correlation table__________p. 7
   Supplementary Table 4: Statistic of pairwise differences between correlations of the synaesthesia screening score with the remaining variables__________p. 8
   Supplementary Table 6: Fit statistics of the full and nested bivariate twin models_p. 9
   Supplementary Table 7: Univariate twin correlations from bivariate models_____p. 10
   Supplementary Table 8: Univariate model results__________p. 10
   Supplementary Table 9: levels of neurodevelopmental / psychiatric features in this and previous population-based studies__________p. 11
4) ***Supplementary References***__________p. 11

1) Additional information about the selected neurodevelopmental / psychiatric feature variables

Autistic features

Autistic features were assessed using an abbreviated self-report version of the autism module of the Autism-Tics, AD/HD, and other Comorbidities inventory (A-TAC)[1]. The answer options were "yes" (scored as 1), "yes, to some extent" (scored as .5), "no" (scored as 0), or "I don't know / don't want to answer" (coded as missing), leading to a maximum possible score of 12. This measure was previously found to predict a clinical autism diagnosis with high accuracy in young adults and to have an acceptable internal consistency[2].

Obsessive-compulsive (OC) features

The Brief Obsessive-Compulsive Scale (BOCS) symptom checklist was used to assess OC symptoms / features[3]. This checklist was developed based on the more comprehensive, clinician-administered Yale-Brown Obsessive-Compulsive Scale[4,5]. The BOCS inquires the life time occurrence of different OC features, and was found to discriminate OCD from other psychiatric disorders with 85% sensitivity and 62-70% specificity[3]. The answer options are "Past" or "Current" (both coded as 1) or "Never" (coded as 0). Like in previous studies using this measure[6], items 14-16 were discarded because they inquire hoarding behaviour, body-dysmorphic concerns and self-harm behaviour, which co-occur with OCD but also frequently occur independent from OC core-symptoms and are considered separate clinical entities[7,8].

Anxiety features

The Screen for Child Anxiety Related Emotional Disorders (SCARED) consists of 38 items assessing anxiety related behaviours and symptoms, for example "When I feel frightened, it is hard for me to breathe". There are three answer possibilities ("Almost never true" coded as 0, "Sometimes true" coded as 1 and "True most of the time" coded as 2). It has been demonstrated to have good convergent validity with other measures of anxiety and internalizing/externalizing behaviours and can to some extent differentiate anxious from non-anxious subjects (Area Under the Curve, AUC = .67) [9].

Depression-related features

The 11-item version of the Center for Epidemiologic Studies Depression Scale (CES-D) [10], a measure widely used in epidemiological settings, was used to assess depression-related features during the past week. The answer options were “Rarely or none of the time” (coded as 1), “Some or a little of the time” (coded as 2), “Occasionally or a moderate amount of time” (coded as 3) and “Most or all of the time” (coded as 4). Of the 11 statements, nine described depression features and two positive states (e.g. “During the past week, I was happy.”) and were hence inverted in the coding.

Psychotic-like experiences (PLE, schizophrenia)

The Psychotic-like Experiences Scale consists of seven items about psychotic-like experiences and thoughts, such as “Sometime I thought that I was being followed or spied upon.” There were four answer options: “Never or very seldom” (coded as 0), “Sometimes” (coded as 1), “Often” (coded as 2) and “Very often” (coded as 3). The screener’s predictive power was good for psychotic-like experiences assessed in a clinical interview (positive predictive value of 100% and a negative predictive value of 88.4%)[11].

ADHD features

ADHD features were assessed using the Adult ADHD Self-Report Scale (ASRS), consisting of 18 items based on DSM-IV symptoms[12,13]. Answer options were ”Never” (coded as 0), ”rarely” (coded as 1), ”Sometimes” (coded as 2), ”Often” (coded as 3) and ”Very often” (coded as 4). All items were summed to create an overall ADHD feature score including both inattention and hyperactivity. A previous study in young adults found that both the inattention and hyperactivity sub-scales of this measure predicted a clinical ADHD diagnosis with high accuracy[2].

(Hypo-)mania-related features (bipolar disorder)

The 13-item self-report Mood Disorder Questionnaire (MDQ) was used to assess features related to (hypo-)mania, such as elevated mood, irritability, and high energy levels. The answer options were “True” (coded as 1) and “Not true (coded as 0). The MDQ is the most widely used screening instrument for bipolar disorder[14].

Eating disorder-related features

Eating disorder related features were assessed using the Eating Disorder Inventory - 2 (EDI-2)[15], which includes 22 statements describing thoughts and behaviours related to eating disorders. The answer options were "Always" (coded as 5), "Usually" (coded as 4), "Often" (coded as 3), "Sometimes" (coded as 2), "Rarely" (coded as 1) and "Never" (coded as 0). The measure has been found to reliably discriminate females with eating disorder symptoms from controls without such symptoms, while its ability to detect anorexia nervosa was limited[15].

2) Control variable Results

Body mass index (BMI, self-reported weight in kilograms divided by self-reported height in meters squared) was used as a control variable where no association with synaesthesia was expected. This was done in order to compare the correlations of interest to the correlation with the control variable as a reference, thereby gaining some insight regarding how easily random correlations reach significance in our sample. We excluded individuals from analyses regarding BMI whose self-reported weight or height were regarded as outliers / errors (132 individuals who indicated to be shorter than 120 cm and one individual who indicated to weigh more than 200 kg), leading to BMI from 5011 individuals to be included (mean / sd ALL = 22.14 / 3.42; mean / sd MZ = 22.06 / 3.53; mean / sd DZ = 22.18 / 3.37).

BMI correlated negatively with the synaesthesia screening score in the randomly selected sub-sample (one twin per pair), however, this correlation was very small and not statistically significant ($r = -.03$, $p=.114$). All other correlations were positive, statistically significant and substantially larger, which we interpret as evidence that the observed correlations did not simply arise due to the sample size (see **Supplementary Table 2**).

3) Supplementary Tables

Supplementary Table 1: More detailed version of Table 1 on previous findings regarding synaesthesia and neurodevelopmental and psychiatric conditions

Condition	First-author	Year	Synaesthesia assessment	mental health measure	association	sample size per group
**Autism**	Baron-Cohen S	2013	survey	clinical diagnosis	+	164 AUT + 97 controls
	Neufeld J	2013	tested	clinical diagnosis	+	21 AUT
	Hughes J	2017	tested	clinical diagnosis	+ (only those with savant)	40 AUT with savant + 34 AUT without savant +29 NT
	Nugent M	2022	tested	self-reported diagnosis	+	282 SYN + 281 non-SYN
	Carmichael DA (experiment 1)	2019	tested	self-reported diagnosis	+	3895 general population (55% female, 98 verified SYN)
	Carmichael DA (experiment 2)	2019	tested	self-reported diagnosis	none	376 via add (90.4% female, 112 verified SYN)
	Ward J	2017	tested	traits	+	35 SYN + 78 AUT
	Ward J	2018	tested	traits	+	182 SYN +189 non-SYN
	Van Leeuwen TM	2019	tested	traits	+	79 SYN + 76 non-SYN
	Taylor M	2023	survey	traits	+	4262 twins from the general population
	Burghoorn F	2020	consistency	traits	+	36 University students (all non-SYN)
	van Leeuwen TM	2021	consistency	traits	+	65 twins (4 SYN)
	Tilot AK	2019	tested	polygenic scores	none	723 SYN + 2181 non-SYN
**Schizophrenia / schizotypy / psychosis**	Nugent, M	2022	survey	self-reported diagnosis	none	282 SYN + 281 non-SYN
	Banissy, MJ	2012	tested	traits	+	30 SYN +30 non-SYN
	Janik McErlean AB	2016	tested	traits	+	35 Syn + 35 non-SYN
	Simmonds-Moore, CA	2019	survey	traits	+/- Sub-scale dependent	1,789 general population
	Tilot AK	2019	tested	polygenic scores	+	723 SYN + 2181 non-SYN
**anxiety**	Carmichael DA	2019	tested	self-reported diagnosis	+	3895 general population (of which 98 verified SYN)
	Simner J	2021	tested	traits	+	76 SYN +1000 non-SYN children
**OCD**	Carmichael DA	2019	tested	self-reported diagnosis	Experiment-dependent	3895 general population (of which 98 verified SYN)
	Wendler E	2019	survey	traits	+	210
**PTSD**	Hoffman SN	2012	One question	self-report screening	+	700 military veterans
	Hoffman SN	2019	One question	self-report screening	+	1,730 military veterans
**Reduced affect**	Simner J	2021	tested	self-reported affect	+	76 SYN +1000 non-SYN children
**ADHD, depression, dyslexia, insomnia**	Carmichael DA	2019	tested	self-reported diagnosis	none	3895 general population (of which 98 verified SYN)

Note: *Synaesthesia assessment: tested = objectively verified synaesthesia based on colour-picker test, survey = self-reported via synaesthesia questionnaire, single question = only one question about synaesthesia was asked; mental health measures: clinical diagnosis = fulfilling diagnostic criteria within clinical assessment, traits = trait /symptom measures related to the diagnostic category; none = association not statistically significant; SYN = number of synesthetic study participants, non-SYN = number study participants who were found not to have the tested or inquired synaesthesia type(s).*

Supplementary Table 2: Pearson correlations between synaesthesia screening score and neurodevelopmental / psychiatric features

	correlation (95% CI)	p	df
**OC features**	.28 (.24 - .31)	< .0001	2540
**Anxiety features**	.23 (.19 - .26)	< .0001	2560
**Depression-related features**	.21 (.18 - .25)	< .0001	2563
**PLE features**	.18 (.14 - .22)	< .0001	2413
**ADHD features**	.19 (.16 -.23)	< .0001	2555
**Autistic features**	.16 (.12 - .20)	< .0001	2569
**Eating disorder-related features**	.15 (.11 - .19)	< .0001	2562
**(Hypo-)mania features**	.15 (.11 - .18)	< .0001	2483
**BMI**	-.03 (-.07 - .01)	.114	2498

Note: *Statistical values from Pearson correlations between synaesthesia screening score and the remaining variables, in half of the sample (one randomly selected twin per pair). Lower bound = lower boundary of the 95% confidence interval, Upper bound = upper boundary of the 95% confidence interval.*

Supplementary Table 3: Full correlation table

	SYN	AUT	OC	ANX	DEP	PLE	ADHD	EAT	MAN
**SYN**	1								
**AUT**	**0.16*** **	1							
**OC**	**0.27*** **	**0.41*** **	1						
**ANX**	**0.22*** **	**0.50*** **	**0.48*** **	1					
**DEP**	**0.22*** **	**0.44*** **	**0.38*** **	**0.66*** **	1				
**PLE**	**0.17*** **	**0.36*** **	**0.36*** **	**0.29*** **	**0.29*** **	1			
**ADHD**	**0.19*** **	**0.45*** **	**0.32*** **	**0.44*** **	**0.47*** **	**0.29*** **	1		
**EAT**	**0.15*** **	**0.27*** **	**0.30*** **	**0.51*** **	**0.54*** **	**0.20*** **	**0.34*** **	1	
**MAN**	**0.13*** **	**0.33*** **	**0.32*** **	**0.24*** **	**0.33*** **	**0.33*** **	**0.41*** **	**0.24*** **	1
**BMI**	-0.03	**0.07** **	0.03	0.01	**0.04*** **	0.03	**0.11*** **	**0.32*** **	0.1

Note: *Pearson r-values for all variables' correlations with each other in half of the sample (one randomly selected twin per pair), significant correlations in* ***bold*** *(* = p < .05; ** = p < .01; *** = p < .001). SYN = Synaesthesia screening score, AUT = Autistic features, ANX = Anxiety features, OC = OC features, PLE = Psychotic-like experiences features, MAN = (hypo-)mania related features, ADHD = ADHD features, EAT = Eating disorder related features, DEP = Depression-related features, BMI = body mass index*

Supplementary Table 4: Statistic of pairwise differences between correlations of the synaesthesia screening score with the remaining variables

	AUT	OC	ANX	DEP	PLE	ADHD	EAT
**OC**	**-5.47*** **						
**ANX**	**-3.37*** **	**2.78** **					
**DEP**	**-2.53* **	**3.09** **	.82				
**PLE**	-.74	**4.58*** **	1.01	1.34			
**ADHD**	-1.57	**3.94*** **	1.77	1.24	-.52		
**EAT**	.81	**5.59*** **	**4.01*** **	**3.27** **	1.38	1.74	
**MAN**	1.09	**5.98*** **	**3.52*** **	**3.16** **	1.89	**2.14* **	.34

Note: *Pearson and Filon's z scores for pair-wise comparisons of Pearson correlations (performed using the R-package cocor) in half of the sample (one randomly selected twin per pair), significant differences in* ***bold*** *(* = p < .05; ** = p < .01; *** = p < .001). AUT = Autistic features, ANX = Anxiety features, OC = OC features, PLE = Psychotic-like experiences features, MAN = (hypo-)mania related features, ADHD = ADHD features, EAT = Eating disorder related features, DEP = Depression-related features.*

Supplementary Table 5: Twin model assumptions testing

Base model	Test model	N Para-meters	df	minus2LL	diff LL	diff df	p
Synaesthesia screening score							
Sat	Sat2	8	346	9585.82	0.35	2	.84
Sat	Sat3	7	346	9586.59	1.12	3	.77
Sat	Sat4	5	346	9588.08	2.61	5	.76
Sat	Sat5	4	347	9589.07	3.60	6	.73
OC features							
Sat	Sat2	8	343	9608.75	4.29	2	.12
Sat	Sat3	7	343	9608.76	4.30	3	.23
Sat	Sat4	5	343	9609.29	4.83	5	.44
Sat	Sat5	4	343	9609.31	4.85	6	.56
Anxiety features							
Sat	Sat2	8	345	9707.08	0.53	2	.77
Sat	Sat3	7	345	9707.16	0.61	3	.90
Sat	Sat4	5	345	9708.37	1.83	5	.87
Sat	Sat5	4	346	9708.38	1.83	6	.94
Depression features							
Sat	Sat2	8	344	9611.42	.70	2	.70
Sat	Sat3	7	345	9624.90	14.18	3	**<.01**
Sat	Sat4	5	345	9625.58	14.86	5	**<.05**
Sat	Sat5	4	345	9632.67	21.95	6	**<.01**
PLE features							
Sat	Sat2	8	345	9734.14	3.13	2	.21
Sat	Sat3	7	346	9734.51	3.49	3	.32
Sat	Sat4	5	346	9735.18	4.16	5	.53
Sat	Sat5	4	346	9736.25	5.24	6	.51
ADHD features							
Sat	Sat2	8	346	9566.76	1.25	2	.54
Sat	Sat3	7	346	9571.71	6.20	3	.10
Sat	Sat4	5	346	9572.90	7.38	5	.19
Sat	Sat5	4	346	9573.79	8.28	6	.22
Autistic features							
Sat	Sat2	8	326	9102.57	0.48	2	.79
Sat	Sat3	7	326	9103.47	1.38	3	.71
Sat	Sat4	5	326	9104.78	2.69	5	.75
Sat	Sat5	4	326	9106.06	3.97	6	.68
Eating disorder related features							
Sat	Sat2	8	345	9605.71	0.47	2	.79
Sat	Sat3	7	345	9612.20	6.96	3	.07
Sat	Sat4	5	345	9613.84	8.60	5	.13
Sat	Sat5	4	345	9615.86	10.62	6	.10
(Hypo-)mania related features							
Sat	Sat2	8	334	9349.75	1.37	2	.51
Sat	Sat3	7	334	9354.43	6.05	3	.11
Sat	Sat4	5	334	9356.28	7.90	5	.16
Sat	Sat5	4	334	9357.75	9.37	6	.15

Note. *Twin model assumptions testing based on model fit comparisons from univariate models. Sat = fully saturated model, in Sat2, means were constrained to be equal across twin 1 and 2 and in Sat3 additionally the variances. In Sat4 and Sat5, means and variances, respectively, were additionally constrained to be equal across zygosity groups. Note that for assumptions testing, the DZ twins were restricted to same-sex twins because opposite and same sex DZ twins differed in mean synaesthesia scores. df = degrees of freedom; minus2LL = minus 2*log-likelihood of the comparison model; diff LL = difference in minus 2*log-likelihoods of the base and comparison model; diff df = difference in degrees of freedom between base and comparison model; p = p-value for likelihood ratio test based on difference in -2LL and difference in df values.*

Supplementary Table 6: Fit statistics of the full and nested bivariate twin models

**Base model**	**Test model**	**N Para-meters**	**df**	**minus2LL**	**diff in LL**	**diff in df**	**p**
OC features * Synaesthesia							
Sat	ACE	11	10209	28166.32	16.74	17	.47
**ACE**	**AE**	**8**	**10212**	**28166.32**	**.00**	**3**	**1.00**
ACE	CE	8	10212	28262.47	96.14	3	<.001
ACE	E	5	10215	28683.11	516.78	6	<.001
**Anxiety *features * Synaesthesia***							
Sat	ACE	11	10257	28391.91	25.47	17	.09
**ACE**	**AE**	**8**	**10260**	**28391.91**	**.00**	**3**	**1.00**
ACE	CE	8	10260	28511.38	119.47	3	<.001
ACE	E	5	10263	28961.88	569.97	6	<.001
PLE features * Synaesthesia							
Sat	ACE	11	10264	28521.35	25.77	17	.08
**ACE**	**AE**	**8**	**10267**	**28521.35**	**.00**	**3**	**1.00**
ACE	CE	8	10267	28616.55	95.20	3	<.001
ACE	E	5	10270	29016.61	495.26	6	<.001
ADHD features * Synaesthesia							
Sat	ACE	11	10273	28382.54	26.60	17	.06
**ACE**	**AE**	**8**	**10276**	**28382.54**	**-8.51**	**3**	**1.00**
ACE	CE	8	10276	28531.81	149.27	3	3.78
ACE	E	5	10279	29048.06	665.53	6	1.69
Autistic features * Synaesthesia							
Sat	ACE	11	9948	27572.69	22.45	17	.17
**ACE**	**AE**	**8**	**9951**	**27572.69**	**.00**	**3**	**1.00**
ACE	CE	8	9951	27666.48	93.78	3	<.001
ACE	E	5	9954	28104.53	531.84	6	<.001
Eating disorder-related features * Synaesthesia							
Sat	ACE	11	10254	28434.55	36.59	17	.004
**ACE**	**AE**	**8**	**10257**	**28434.55**	**.00**	**3**	**1.00**
ACE	CE	8	10257	28599.85	165.30	3	<.001
ACE	E	5	10260	29087.66	653.11	6	<.001
(Hypo-)mania-related features * Synaesthesia							
Sat	ACE	11	10083	28025.23	18.63	17	0.35
**ACE**	**AE**	**8**	**10086**	**28025.23**	**.00**	**3**	**1.00**
ACE	CE	8	10086	28118.87	93.64	3	<.001
ACE	E	5	10089	28514.64	489.40	6	<.001

Note. Comparisons between fully saturated, ACE and nested bivariate models. *Synasethesia = Synaesthesia screening score; A = additive genetics; C = shared environments; E = non-shared environment; df = degrees of freedom; minus2LL = minus 2*log-likelihood of the comparison model; diff LL = difference in minus 2*log-likelihoods of the base and comparison model; diff df = difference in degrees of freedom between base and comparison model; p = p-value for likelihood ratio test based on difference in -2LL and difference in df values.*

Supplementary Table 7: Univariate twin correlations from bivariate models

	MZ	DZ
Synaesthesia	.49 (.44 - .54)	.22 (.17 - .26)
OC	.43 (.37 - .48)	.18 (.13 - .23)
Anxiety	.47 (.42 - .52)	.18 (.13 - .23)
Depression	.48 (.43 - .53)	.19 (.15 - .24)
PLE	.39 (.34 - .44)	.16 (.11 – 21)
ADHD	.54 (.50 - .58)	.20 (.16 - .25)
Autism	.43 (.38 - .49)	.20 (.16 - .25)
Eating	.56 (.51 - .60)	.17 (.12 - .21)
(Hypo-)mania	. 40 (.34 – .46)	. 16 (.11 - .20)

***Note. Twin correlations** (how much each trait correlates between twins of a pair) in sub-samples of monozygotic (MZ) and dizygotic (DZ) twins, extracted from the constrained, saturated bivariate models. Synaesthesia = Synaesthesia screening score, OC = obsessive-compulsive features, Anxiety = anxiety features, Depression = Depression-related features, PLE = psychotic-like experiences, ADHD = ADHD features, Eating = Eating disorder-related features, (Hypo-)mania = (Hypo-)mania-related features.*

Supplementary Table 8: Univariate model results

	A (95% CI)	E (95% CI)	MZ	DZ
Synaesthesia	.48 (.44 - .52)	.52 (.48 – .57)	.49 (.44 - .55)	.22 (.17 - .27)
OC	.41 (.36 -.46)	.59 (.54 - .64)	43 (.37 -. 49)	.18 (.13 – .23)
Anxiety	.45 (.40 - .49)	.55 (.51 - .60)	.47 (.41 - .53)	.18 (.13 - .23)
Depression	.46 (.41 - .51)	.54 (.49 - .59)	.49 (.43 - .54)	.20 (.15 - .24)
PLE	.38 (.32 - .42)	.63 (.58 - .68)	.39 (.33 - .45)	.16 (.11 - .21)
ADHD	.51 (.47 - .56)	.49 (.44 - .53)	.54 (.49 - .59)	.20 (.15 - .25)
Autism	.43 (.38 - .48)	.57 (.52 - .62)	.44 (.38 - .50)	.21 (.15 - .36)
Eating	.51 (.47 - .56)	.49 (.45 - .54)	.56 (.51 - .61)	.17 (.12 – .21)
(Hypo-)mania	.38 (.33 - .43)	.62 (.57 - .67)	.41 (.34 -.47)	.16 (.11 – .20)

***Note. Variance components** from univariate twin models and **twin correlations** in sub-samples of monozygotic (MZ) and dizygotic (DZ) twins, extracted from the constrained, saturated univariate models. A = additive genetic component, E = non-shared environment component, CI = confidence interval, Synaesthesia = Synaesthesia screening score, OC = obsessive-compulsive features, Anxiety = anxiety features, Depression = Depression-related features, PLE = psychotic-like experiences, ADHD = ADHD features, Eating = Eating disorder-related features, (Hypo-)mania = (Hypo-)mania-related features.*

Supplementary Table 9: levels of neurodevelopmental / psychiatric features in this and previous population-based studies

	**Mean / SD this sample**	**Previous study mean (sd) / age or age range in years / Reference**
**OC**	2.29 (2.51)	1.82 (2.24) / 18 / Krebs et al., 2021[6]
**Anxiety**	18.33 (12.56)	21.5 (12.4) / 8-12 / Wren et al., 2007[16]
**Depression**	19.66 (6.07)	17.0 (10.0) / # / Carpenter et al., 1998[10]
**PLE**	.98 (1.57)	1.13 (1.85) / 16 / Taylor et al., 2022[17]
**ADHD**	24.89 (12.92)	30.52 (9.2) / 18-69 / Panagiotidi et al., 2018[18]
**Autistic**	1.92 (1.64)	.73 (1.07) / 18 / Taylor et al., 2017[19]
**Eating**	32.11 (20.38)	29.1 (19.9)* or 53.4 (28.7)** / 18-24 / Nevonen & Broberg, 2001[15]
**(Hypo-)mania**	4.80 (3.52)	7.16 (2.62) / 16 / Gonzales-Calvo et al., 2024[20]

Note. *Means and standard deviations of the psychiatric / neurodevelopment feature measures used in our study and previous population-based studies. Observe that our sample was assessed at the age of 18, while the majority of the previous studies assessed different ages and wider age ranges, which might explain the discrepancies; #3rd year undergraduate female students – age wasn't reported; *female controls without eating problems (n=188); **female controls with eating problems (n=51). OC = obsessive-compulsive features, Anxiety = anxiety features, Depression = Depression-related features, PLE = psychotic-like experiences, ADHD = ADHD features, Eating = Eating disorder-related features, (Hypo-)mania = (Hypo-)mania-related features.*

5) Supplementary References

1. Larson T, Anckarsäter H, Gillberg C, Ståhlberg O, Carlström E, Kadesjö B, et al. (2010) The autism-tics, AD/HD and other comorbidities inventory (A-TAC): further validation of a telephone interview for epidemiological research. *BMC Psychiatry* 10:1-11.
2. Ghirardi L, Pettersson E, Taylor MJ, Freitag CM, Franke B, Asherson P, et al. (2019) Genetic and environmental contribution to the overlap between ADHD and ASD trait dimensions in young adults: a twin study. *Psychological Medicine*;49(10):1713-21.
3. Bejerot S, Edman G, Anckarsäter H, Berglund G, Gillberg C, Hofvander B, et al. (2014) The Brief Obsessive–Compulsive Scale (BOCS): A self-report scale for OCD and obsessive–compulsive related disorders. *Nordic Journal of Psychiatry*;68(8):549-59.
4. Goodman WK, Price LH, Rasmussen SA, Mazure C, Delgado P, Heninger GR, et al. (1989) The Yale-Brown Obsessive Compulsive Scale: II. Validity. *Archives of General Psychiatry;*46(11):1012-6.
5. Goodman WK, Price LH, Rasmussen SA, Mazure C, Fleischmann RL, Hill CL, et al. (1989) The Yale-Brown Obsessive Compulsive Scale: I. Development, use, and reliability. *Archives of General Psychiatry*;46(11):1006-11.
6. Krebs G, Mataix-Cols D, Rijsdijk F, Rück C, Lichtenstein P, Lundström S, et al. (2021) Concurrent and prospective associations of obsessive-compulsive symptoms with suicidality in young adults: A genetically-informative study. *Journal of Affective Disorders*;281:422-30.

7. Mataix-Cols D, Frost RO, Pertusa A, Clark LA, Saxena S, Leckman JF, et al. (2010) Hoarding disorder: a new diagnosis for DSM-V?. *Depression and Anxiety*;27(6):556-72.
8. Phillips KA, Pinto A, Menard W, Eisen JL, Mancebo M, Rasmussen SA. (2007) Obsessive–compulsive disorder versus body dysmorphic disorder: a comparison study of two possibly related disorders. *Depression and Anxiety*;24(6):399-409.
9. Monga S, Birmaher B, Chiappetta L, Brent D, Kaufman J, Bridge J, et al. (2000) Screen for child anxiety-related emotional disorders (SCARED): Convergent and divergent validity. *Depression and Anxiety*;12(2):85-91.
10. Carpenter J, Andrykowski M, Wilson J, Hall L, Kay Rayens M, Sachs B, et al. (1998) Psychometrics for two short forms of the Center for Epidemiologic Studies-Depression Scale. *Issues in Mental Health Nursing*;19(5):481-94.
11. Kelleher, I. & Cannon, M. (2011) Psychotic-like experiences in the general population: characterizing a high-risk group for psychosis. *Psychological Medicine* 41(1):1-6.
12. Adler LA, Spencer T, Faraone SV, Kessler RC, Howes MJ, Biederman J, et al. (2006) Validity of pilot Adult ADHD Self-Report Scale (ASRS) to rate adult ADHD symptoms. *Annals of Clinical Psychiatry*;18(3):145-8.
13. Kessler RC, Adler L, Ames M, Demler O, Faraone S, Hiripi EV, et al. (2005) The World Health Organization Adult ADHD Self-Report Scale (ASRS): a short screening scale for use in the general population. *Psychological Medicine*;35(2):245-56.
14. Zimmerman M, Galione JN. (2011) Screening for bipolar disorder with the Mood Disorders Questionnaire: a review. *Harvard Review of Psychiatry*;19(5):219-28.
15. Nevonen L & Broberg AG. (2001) Validating the eating disorder inventory-2 (EDI-2) in Sweden. *Eating and Weight Disorders-Studies on Anorexia, Bulimia and Obesity* 6:59-67.
16. Wren FJ, Berg EA, Heiden LA, Kinnamon CJ, Ohlson LA, Bridge JA, et al. (2007) Childhood anxiety in a diverse primary care population: parent-child reports, ethnicity and SCARED factor structure. *Journal of the American Academy of Child & Adolescent Psychiatry*, 46(3):332-340.
17. Taylor MJ, Freeman D, Lundström S, Larsson H and Ronald A. (2022). Heritability of psychotic experiences in adolescents and interaction with environmental risk. *JAMA Psychiatry*, 79(9):889-897, doi:10.1001/jamapsychiatry.2022.1947.
18. Panagiotidi M, Overton PG, Stafford T. (2018) The relationship between ADHD traits and sensory sensitivity in the general population. *Comprehensive Psychiatry*, 80:179-185.
19. Taylor MJ, Gillberg C, Lichtenstein P and Lundström S. (2017). Etiological influences on the stability of autistic traits from childhood to early adulthood: evidence from a twin study. *Molecular Autism*, 8:1-9, DOI 10.1186/s13229-017-0120-5
20. Gonzalez-Calvo I, Ronald A, Shakoor S, Taylor MJ, Eley TC, Hosang GM. (2024) Perinatal risk factors and subclinical hypomania: A prospective community study. *Journal of Affective Disorders*, 362:885-892.
